# Do medical students receive training in correct use of personal protective equipment?

**DOI:** 10.1080/10872981.2017.1264125

**Published:** 2017-01-04

**Authors:** Amrita John, Myreen E. Tomas, Aditya Hari, Brigid M. Wilson, Curtis J. Donskey

**Affiliations:** ^a^ Department of Medicine, Division of Infectious Diseases and HIV Medicine, University Hospitals Case Medical Center, Cleveland, OH, USA; ^b^ Geriatric Research, Education and Clinical Center, Cleveland VA Medical Center, Cleveland, OH, USA; ^c^ Case Western Reserve University School of Medicine, Cleveland, OH, USA

**Keywords:** Ebola, medical students, infection control, training, education

## Abstract

**Background:** Healthcare personnel often use incorrect technique for donning and doffing of personal protective equipment (PPE).

**Objective:** We tested the hypothesis that medical students receive insufficient training on correct methods for donning and doffing PPE.

**Methods:** We conducted a cross-sectional survey of medical students on clinical rotations at two teaching hospitals to determine the type of training they received in PPE technique. The students performed simulations of contaminated PPE removal with fluorescent lotion on gloves and were assessed for correct PPE technique and skin and/or clothing contamination. To obtain additional information on PPE training during medical education, residents, fellows, and attending physicians completed written questionnaires on PPE training received during medical school and on knowledge of PPE protocols recommended by the Centers for Disease Control and Prevention.

**Results:** Of 27 medical students surveyed, only 11 (41%) reported receiving PPE training, and none had received training requiring demonstration of proficiency. During simulations, 25 of 27 (92.5%) students had one or more lapses in technique and 12 (44%) contaminated their skin with fluorescent lotion. For 100 residents, fellows and attending physicians representing 67 different medical schools, only 53% reported receiving training in use of PPE and only 39% selected correct donning and doffing sequence.

**Conclusions:** Our findings suggest that there is a need for development of effective strategies to train medical students in correct use of PPE.

**Abbreviations:** PPE: Personal protective equipment; MRSA: Methicillin-resistant *Staphylococcus aureus*; SARS: Severe acute respiratory syndrome; MERS: Middle East respiratory syndrome; WHO: World Health Organization; CDC: Centers for Disease Control and Prevention; OSCE: Objective structured clinical examination

## Introduction

Effective use of personal protective equipment (PPE) is essential to protect personnel and patients in healthcare settings [1]. However, recent studies suggest that personnel frequently use incorrect technique for donning and doffing PPE [2,3]. Lapses in technique can lead to an increased risk for contamination of skin and clothing with healthcare-associated pathogens [2,4]. Such contamination contributes to transmission of pathogens such as *Clostridium difficile* [5] and methicillin-resistant *Staphylococcus aureus* (MRSA) [1], and places patients and medical personnel at risk for acquisition of infection, including with potentially fatal pathogens such as Middle East respiratory syndrome (MERS) coronaviruses, and Ebola virus [6,7].

One strategy to reduce the risk for contamination during donning and doffing of PPE is to educate personnel on proper technique based on Centers for Disease Control and Prevention (CDC) protocols [8]. However, in a survey of 222 healthcare personnel, we found that training in correct PPE technique was often suboptimal with no requirement for demonstration of proficiency in donning and doffing of PPE [9]. Fourteen percent of physicians reported no previous training in the use of PPE [9]. Ideally, training in the use of PPE and other infection control practices would be included in the medical school curriculum. However, previous studies suggest that medical students may not receive adequate training in infection control practices such as hand hygiene and standard precautions [10–15].

The goal of our study was to test the hypothesis that medical students receive insufficient training on correct methods for donning and doffing PPE. We employed both qualitative (observations, key informant interviews) and quantitative (simulations of contaminated PPE removal, surveys) methods. Medical students from three Northeast Ohio medical schools were interviewed regarding PPE training, and simulations were conducted to assess PPE technique and risk for skin and/or clothing contamination. To further investigate PPE training across a wide range of medical schools, residents, fellows, and attending physicians were surveyed regarding PPE training they received during medical school and on knowledge of PPE protocols recommended by the Centers for Disease Control and Prevention.

## Methods

The study was conducted at two acute-care teaching hospitals in Cleveland between October 2015 and May 2016. The hospitals provided training for medical students from three different medical schools, residents in internal medicine and surgery, and fellows from multiple subspecialties. The institutional review boards of both institutions approved the study protocol.

### Medical student interviews and evaluations of PPE technique

**Figure 1.**
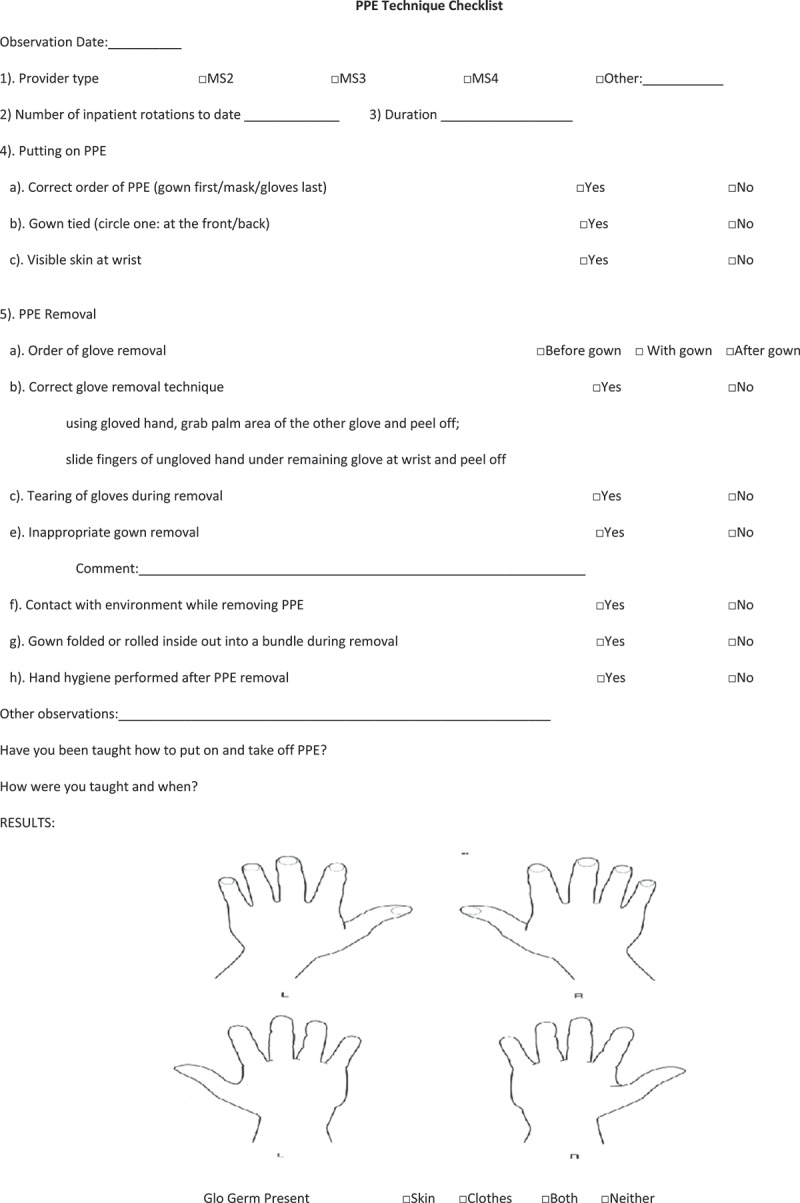
Standardized checklist used for assessment of personal protective equipment (PPE) donning and doffing by medical students.

Medical students were interviewed to obtain information on year of training, number of clinical rotations, and prior training in use of PPE. Students donned and doffed contact isolation gowns and nitrile gloves using their usual technique. After donning gloves, fluorescent lotion was applied to gloved hands. After PPE removal, a black light was used to assess for contamination of the skin of the hands and wrists. Simulations were observed and deviations in technique from CDC recommendations were recorded using a standardized checklist (Figure 1).

### Surveys and interviews of residents, fellows, and attending physicians

A convenience sample of medical residents, fellows, and attending physicians at the two study hospitals was asked to complete a questionnaire that included questions on years of experience in healthcare, name and location of medical school, training in use of PPE during medical school, and questions on the sequence for donning and doffing recommended by the CDC. Questionnaire data was analyzed using R 3.2.2 statistical software. Pearson’s Chi-square test with Yates’ continuity correction was used to compare responses between groups of physicians (US versus non-US trained, Residents versus Fellows/Attendings).

## Results

For 27 medical students participating in the study, the average number of weeks of clinical experience was 24 (range 0–52 weeks). Of the 27 students, 16 (59%) did not recall prior training in the use of PPE, six (22%) completed an online computer-based training module at the start of their clinical rotations, three (11%) received a lecture that included PPE training, and two (7%) reported having a demonstration on how to use PPE. For those completing computer modules, it was noted that the training focused primarily on the type of PPE to be employed in different situations rather than on how to use PPE. None of the students recalled having to demonstrate their proficiency in use of PPE. Several students commented that they did not receive instruction on how to use PPE, but learned by observing other personnel.

During simulations of contaminated glove removal, five students (19%) exhibited correct donning technique and six (22%) exhibited correct doffing technique; only two students (7%) exhibited correct donning and doffing technique. Of the 27 students, 12 (44%) contaminated their hands and/or wrist with fluorescent lotion during glove removal.

Table 1 shows the results of the survey for medical residents versus fellows and attendings. The 100 participants represented 67 different medical schools in 18 countries including the United States (N = 34 medical schools), Columbia, Europe, the Caribbean, India, China, Israel, and the Middle East. A majority of participants reported no training in use of PPE during medical school or residency. Less than 40% of participants chose correct donning and/or doffing sequences based on CDC recommendations. There were no significant differences among physicians trained in United States versus physicians trained in medical schools outside the United States, in terms of having had training in use of PPE (p = 0.46) or their knowledge of the CDC recommendations for donning and/or doffing sequences (p = 0.44). Similarly there were no significant differences in use of PPE training or knowledge between residents and fellows/attendings (p > 0.05 for all comparisons).

**Table 1.** Survey on training and knowledge in the use of personal protective equipment among physicians.

	Residents (n = 69)	Attendings/Fellows (n = 31)
Number of years since graduation from medical school, mean (range); median	2.5 (1–14); 2	16.7 (2–40); 16
**Location of medical school**		
USA	44 (64%)	18 (58%)
Canada	1 (1%)	0 (0%)
Europe	1 (1%)	1 (3%)
Caribbean	8 (12%)	2 (7%)
Asia	8 (12%)	4 (13%)
Middle East	6 (9%)	0 (0%)
South America	0 (0%)	5 (16%)
Unknown	1 (1%)	1 (3%)
**Training in use of personal protective equipment occurred in:**		
Medical school only	16 (23%)	2 (6%)
Residency only	2 (3%)	7 (23%)
Trained in medical school and residency	20 (29%)	6 (19%)
**Knowledge of CDC recommended sequence for donning and doffing**		
Correct donning sequence	32 (46%)	19 (61%)
Correct doffing sequence	40 (58%)	25 (81%)
Both donning and doffing sequence correct	22 (32%)	17 (55%)

## Discussion

Training in correct use of PPE and other basic infection control practices should ideally be an integral part of medical education. However, our findings suggest that training in correct use of PPE is suboptimal or nonexistent in many medical schools. Only 41% of medical students from three schools in Northeast Ohio reported receiving training in PPE use, and none reported a requirement for demonstration of proficiency. In simulations of contaminated glove removal, 7% of students exhibited correct donning or doffing technique and 44% contaminated their hands and/or wrist with fluorescent lotion. The survey of residents, fellows and attendings representing 67 medical schools in 18 countries revealed that training in correct use of PPE was uncommon during medical education with less than 40% of participants choosing the correct donning and/or doffing sequences based on CDC recommendations.

Previous studies have identified deficiencies in knowledge of medical students regarding standard precautions and hand hygiene [10–15]. We are not aware of previous studies that have specifically evaluated training of medical students in correct use of PPE. Given the need to train medical students in a variety of infection control practices, a practical approach may be to incorporate PPE training into general sessions designed to train personnel in infection control measures. Incorporation of fluorescent lotions or powders into PPE training sessions may be valuable to provide immediate visual feedback on routes and sites of contamination [2]. Use of these methods with a requirement for demonstration of proficiency within an Objective structured clinical examination (OSCE) or routine clinical setting may be effective in reducing the risk for contamination [16–18].

In addition to protecting healthcare personnel, correct use of PPE is essential for patient safety. A significant proportion of healthcare delivery has moved from healthcare facilities to outpatient and homecare settings [19,20], and it is not uncommon for patients receiving homecare nursing to be colonized or infected with multidrug-resistant organisms [21]. Healthcare-associated infections have a significant financial impact on the U.S. healthcare system, and many of these infections are considered reasonably preventable [22–24]. Thus, correct use of PPE to prevent pathogen transmission is as important in community healthcare settings as in the hospital. Efforts to improve PPE education should include community nursing personnel.

Our study has some limitations. The medical students were from only three medical schools. However, the surveys of residents, fellows, and attendings from 67 different medical schools clearly suggest that suboptimal PPE training is commonplace in medical education. In addition, there is a possibility of recall bias on the part of the current practicing physicians regarding PPE training during their medical school education. Further studies are needed to evaluate the curriculum of medical schools with regard to training in basic infection control practices such as effective use of PPE.

## Conclusion

Our findings suggest that training in correct use of PPE is insufficient in medical school education. Incorrect use of PPE puts students at risk for acquisition of infection and contribute to transmission of pathogens. Future studies are needed to develop effective strategies to train medical students in correct use of PPE.
